# Sensitization of Human Pancreatic Cancer Cells Harboring Mutated *K-ras* to Apoptosis

**DOI:** 10.1371/journal.pone.0040435

**Published:** 2012-07-25

**Authors:** Ling Shen, Sung-Hoon Kim, Chang Yan Chen

**Affiliations:** 1 Department of Radiation Oncology, Beth Israel Deaconess Medical Center, Harvard Medical School, Boston, Massachusetts, United States of America; 2 Lab of Angiogenesis and Chemoprevention, Graduate School of East-West Medical Science, Kyunghee University, Seoul, South Korea; Wayne State University School of Medicine, United States of America

## Abstract

Pancreatic cancer is a devastating human malignancy and gain of functional mutations in *K-ras* oncogene is observed in 75%–90% of the patients. Studies have shown that oncogenic *ras* is not only able to promote cell growth or survival, but also apoptosis, depending upon circumstances. Using pancreatic cancer cell lines with or without expressing mutated *K-ras*, we demonstrated that the inhibition of endogenous PKC activity sensitized human pancreatic cancer cells (MIA and PANC-1) expressing mutated *K-ras* to apoptosis, which had no apoptotic effect on BxPC-3 pancreatic cancer cells that contain a normal Ras as well as human lung epithelial BAES-2B cells. In this apoptotic process, the level of ROS was increased and PUMA was upregulated in a p73-dependent fashion in MIA and PANC-1 cells. Subsequently, caspase-3 was cleaved. A full induction of apoptosis required the activation of both ROS- and p73-mediated pathways. The data suggest that PKC is a crucial factor that copes with aberrant *K-ras* to maintain the homeostasis of the pancreatic cancer cells harboring mutated *K-ras*. However, the suppression or loss of PKC disrupts the balance and initiates an apoptotic crisis, in which ROS and p73 appear the potential, key targets.

## Introduction

Pancreatic cancer is a disease with a dismal outlook. In the United States, approximately 33,000 patients are diagnosed with pancreatic cancer each year and almost an equal number will die from this malignant disease [1]–[3]. Worldwide, pancreatic cancer causes a significant numbers of deaths annually. Several features of this devastating disease are responsible for the high rate of mortality, due to the difficulty to detect precursor lesions or noticing the symptoms until in the advanced stages. The cancer often undergoes micro-metastasis, which is responsible for a poor prognosis of this disease. In addition, advanced pancreatic cancer is often resistant to conventional chemotherapy or radiotherapy. All of these indicate the urgency for developing new strategies to treat this fatal disease.

Genetic, epigenetic and environmental factors are involved in the genesis and development of pancreatic cancer [1]. The pathogenesis of this disease is through accumulation of genetic and molecular changes, resulting in defects in the growth, adhesion and integration of the pancreases. The molecular alterations in this human malignancy have been shown to include changes in growth-related molecules, tumor suppressors and cell cycle regulators [4]–[7]. Disturbances in intracellular, mitogenic signaling pathways or proliferation restrictions provide an advantage to tumor cells for their growth or survival. Epidermal growth factor receptor (EGFR) family is the plasma membrane glycoprotein and was shown to play a crucial role in pancreatic cancer initiation and development [8]–[10]. The members of EGFR have been reported to be often overexpressed in pancreatic cancer cells, which accordantly activated its downstream signaling pathways, such as Ras and ERK, to promote cell growth and survival [11].

Although a variety of genetic aberrant markers have been identified in human pancreatic cancer lesions, the most frequent mutations were identified in *K-ras*, and the closely following events were the inactivation of the tumor suppressors of p16, ARF, p53 and SMAD4 [12]–[14]. K-Ras belongs to the Ras family proteins that are small cytoplasmic GTP binding proteins [15]. The constitutively active Ras associates with GTP, and confers uncontrollable, mitogenic-stimulatory signaling to downstream effectors, including Raf/MAPK, PI3K/Akt, JNK/p38 and RalGDS. A mouse model of pancreatic cancer showed that conditional expression of an oncogenic allele of *K-ras* was able to form pre-ductal lesions that progressed to invasive and metastatic cancer at a low frequency. Concurrent knockout of p14 and ARF promoted and transformed these pre-lesions to highly invasive and metastatic cancers [12]–[14]. These results indicate that K-Ras activation induces pre-pancreatic lesions and the tumor suppressors (such as p14 or ARF) function to restrict the malignant conversion of these precursors [12]–[14]. However, it has not been fully explored if these intracellular pathways in pancreatic cancer cells can be re-directed to switch on cell death program.

It is well known that Ras can promote not only cell proliferation or differentiation, but also programmed cell death. In APO1-mediated apoptosis, the ligation with APO1 (apoptosis antigen 1) receptor caused the accumulation of membrane lipids and activation of ceramide, which in turn, stimulate Ras activity for the induction of apoptosis [16], [17]. In lymphocytes, Ras played an important role in IL-2- mediated apoptosis, which ensured effective turnover of lymphocytes [18], [19]. Abrupt activation of Ras downstream effector MAP kinase pathway promoted cells to undergo apoptosis [20], [21]. In response to stress-related stimulation, JNK appeared to function at downstream of Ras and induce apoptosis in cells when stress was persistent [22]. We reported that oncogenic Ha-Ras sensitized human or murine cells to apoptosis when endogenous PKC activity is suppressed [22]. In this apoptotic process, the level of ROS was increased and caspase cascade was triggered [22]. Our present study aimed at further testing whether *K-ras* mutation was synthetically lethal with loss of PKC in pancreatic cancer cells.

PKC (protein kinase C) family consists of more than 11 isoforms that are classified on the basis of their biochemical functions and structures into the classical (cPKCs: α, β and γ that are phorbol ester and calcium-dependent), novel (nPKCs: δ, ε, η and θ that are phobol ester-dependent only) and atypical PKCs (aPKCs: ζ and λ that are independent of phorbol ester and calcium). Mitogenic stimuli (such as growth factors), through increasing the membrane DAG (diacylglycerol), activate PKC. While studies have shown that PKC was involved in phorbol ester-mediated mitogenic responses, it is now clear that PKC activation could inhibit cell growth or even trigger apoptosis, depending upon types of the isoforms, differential coupling to effectors [23], [24]. For example, PKC α often mediates proliferative or tumorigenic responses. In intestinal or mammary cells, the same isoforms of PKC participate in anti-proliferative responses. However, different PKC isoforms in the same type of cells could function oppositely. In murine NIH3T3, rat R6 or normal human colonic epithelial cells, overexpression of PKC δ caused growth arrest while increasing level of PKC ε initiated transformation process [25]–[27]. Emerging evidence strongly suggested that PKC δ often acts as a tumor suppressor [24], [28]. Studies showed that PKC δ not only was a negative regulator of the cell cycle progression or positive mediator of apoptosis, but also rendered a high resistance to skin tumor promotion induced by DMBA-phorbol ester in animal models [29], [30].

The crosstalk between PKCs and Ras signaling pathways has been observed [31]. In different types of cells, PKC and Ras interact either in a hierarchic linear or cooperative parallel relationship. In response to mitogenic stimulation, PKC was phosphorylated at various serine residues and subsequently associated with the SH2 domain of Grb-2. The complex including Grb-2/Sos was, in turn, formed to activate Ras signaling in T lymphocytes [32]. The activation of PKC and Ras in lymphocytes was then able to mobilize PI3 kinase to generate PIP3 and further cause various protein kinase cascades, leading to the activation of AKT and Rac to promote cell growth-related activities. It was also reported that through affecting Rel activity, PKC had a negative influence on Ras-mediated signaling [31]. In certain malignant cells, PKC was activated and able to mediated Bcl-2 phosphorylation for survival promotion [33], [34]. By blocking pro-apoptotic signaling pathways, PKC-induced activation of Bcl-2 was suggested to play a significant role in lung tumorigenesis. Inhibition of PKC by pharmacological inhibitors in cultured human lung cancerous cells triggered an apoptotic response [35]. Our current study demonstrated that Ras preferentially induced apoptosis in pancreatic cancer cells harboring an active *K-ras* after the blockade of PKC. Our data suggested that the cooperation of PKC appeared crucial for pancreatic cancer cells harboring mutated K-Ras to survive.

## Results

### Sensitization of Pancreatic Cancer Cells Harboring Mutated K-ras to Apoptosis after the Treatment with GO6976

**Figure 1:**
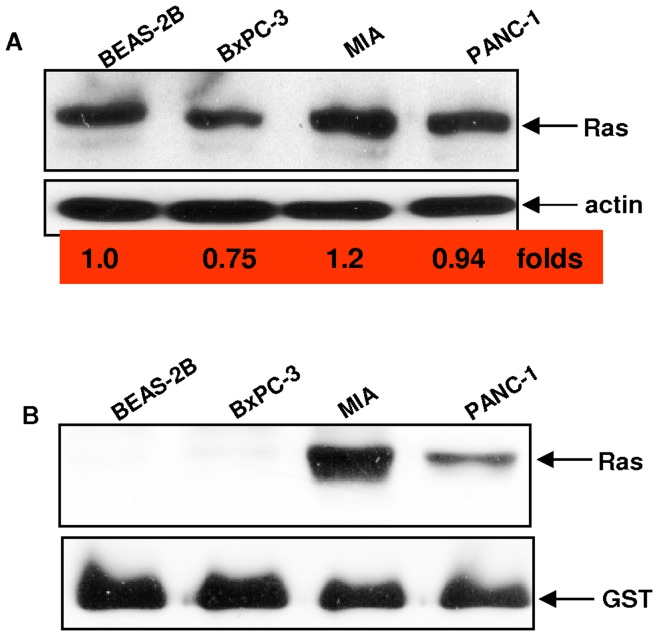
Activation status of Ras in pancreatic cancer cells. **A.** The expression of Ras was examined by immunoblotting analysis in human pancreatic cancer BxPC-3, MIA, PANC-1 or lung epithelial BEAS-2B cells. The folds of the expression levels of Ras in pancreatic cancer cells relative to that in BEAS-2B cells were measured and indicated. Equal loading of total proteins per lane was determined by β-actin. **B.** Ras GTP-binding activity was measure in these cells by Ras-GTP assay. The blot was re-probed by anti-Ras antibody to judge evenly loading of total proteins.

The duel functions of Ras to promote cell growth and apoptosis was well documented [20], [22]. Previously, we demonstrated that the co-inhibition of PKC α and β expression or activity was lethal to murine cells overexpressing *v-ras* [22], [28]. Since *K-ras* mutations are detected in most of pancreatic adenocarcinoma lesions [1], it led us to examine the susceptibility of pancreatic cancer cells to apoptosis in response to the treatment with GO6976 (a PKC inhibitor specific for PKC α and β). The expression and activation status of K-Ras in different human pancreatic cancer cell lines and human lung epithelial cells were tested. The level of Ras expression in human pancreatic cancer MIA or PANC-1 cells was comparable with that in lung epithelial BEAS-2A cells and a slightly reduced amount of Ras was detected in pancreatic cancer BxPC-3 cells (**Fig. 1A**). Subsequently, the lysates from these cells were precipitated with a RBD (Ras binding domain of Raf)-GST fusion protein to test the activation status of Ras. An activated Ras was co-precipitated with the fusion protein in MIA and PANC-1 cells, but not in BxPC-3 or BEAS-2B cells (**Fig. 1B**). The magnitude of Ras activation in MIA was higher than that in PANC-1 cells.

**Figure 2:**
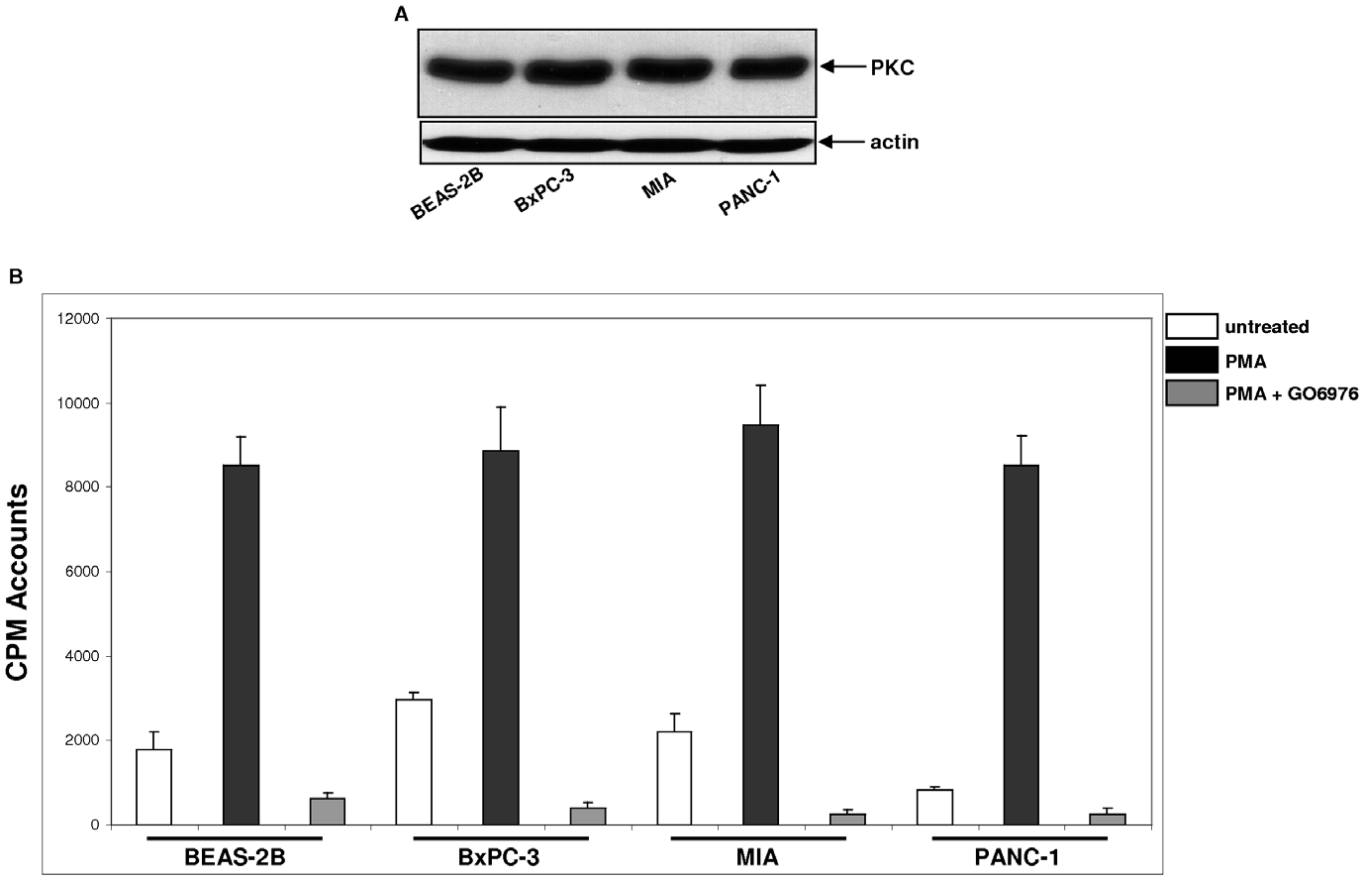
PKC expression and activity following PMA or PMA plus GO6976 treatment. **A.** Cell lysates isolated from untreated cells were immunoblotted with anti-pan-PKC antibody. *β*-actin was used to determine equal loading of total protein per lane. **B.** Cell lysates from the cells with or without being treated with PMA or PMA plus GO6976, were immunoprecipitated with anti-pan-PKC antibody. The immunocomplexes were incubated with [^32^P] γ-ATP and the peptide substrates to analyze PKC activity. The error bars represented SD from three independent experiments (n = 3, *ρ*<0.05).

Next, we examined the expression of PKC in these cells as well as their responses to PKC activator PMA (phorbol myristate acetate) or inhibitor GO6976. A comparable expression level of PKC was detected in all the cell lines (**Fig. 2A**). The induction PKC activity by PMA and inhibitory effect of GO6976 on PMA-induced PKC activity were also examined using a PKC kinase activity kit (**Fig. 2B**). In untreated MIA or PANC-1 cells, PKC activity was slightly higher than that in untreated BxPC-3 or BEAS-2B cells. The treatment with PMA dramatically increased the activity of this kinase in all cell lines, which was inhibited by GO6976, indicating that PKC was functional in all cells tested.

**Figure 3:**
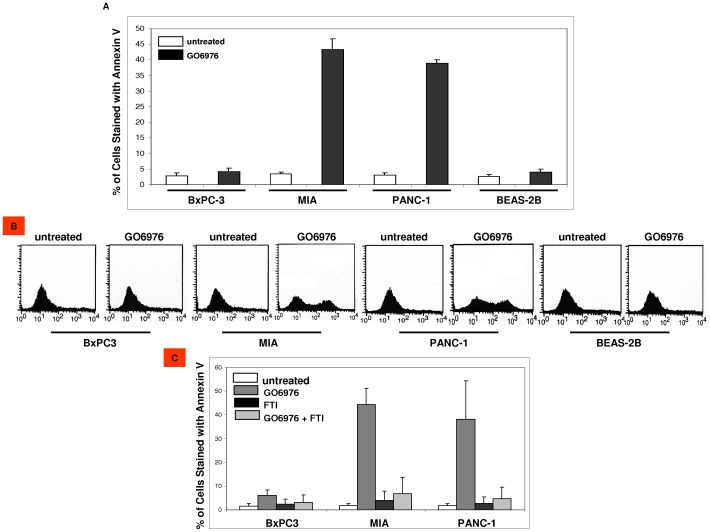
Induction of apoptosis in GO6976-treated pancreatic cancer cells. **A.** Cells were treated with GO6976 (1 µM) for 48 h and then collected for annexin V assay. The error bars are SD from 5 independent experiments (n = 5, *ρ*<0.05). **B.** Profiles of the cells after being stained with annexin V. **C.** Cells were treated with FTI for 30 min prior to GO6976 treatment. Subsequently, annexin V assay was conducted. The error bars are SD from 5 independent experiments (n = 5, *ρ<*0.05).

PKC and Ras are the crucial intracellular signal transducers and participate in transmitting signals to regulate various biological or patho-biological activities. The induction of apoptosis by PKC suppression was reported in murine or human cells expressing oncogenic *ras* [20], [22], [28]. To test whether GO6976 was able to induce apoptosis in pancreatic cancer cell lines with or without expressing mutated *K-ras*, annexin V assay was performed (**Fig. 3A and B**). GO6976 treatment dramatically sensitized MIA and PANC-1 cells to apoptosis and played no role in the induction of apoptosis in BxPC-3 cells that contain normal Ras signaling as well as in human lung epithelial BEAS-2B cells. Notably, MIA cells in which Ras activity was higher were more susceptible to GO6976-induced apoptosis than PANC-1 cells. To determine if Ras was responsible for the induction of apoptosis, farnesyltransferase inhibitor (FTI) to suppress Ras activity was used (**Fig. 3C**). In the presence of FTI, GO6976 treatment was unable to induce apoptosis in MIA or PANC-1 cells. The data suggested that mutated K-Ras, together with loss of PKC function, appeared lethal and this lethal reaction might be regulated by mutated Ras.

### Upregulation of ROS by GO6976 in Pancreatic Cancer Cells Harboring Mutated K-ras for the Induction of Apoptosis

**Figure 4:**
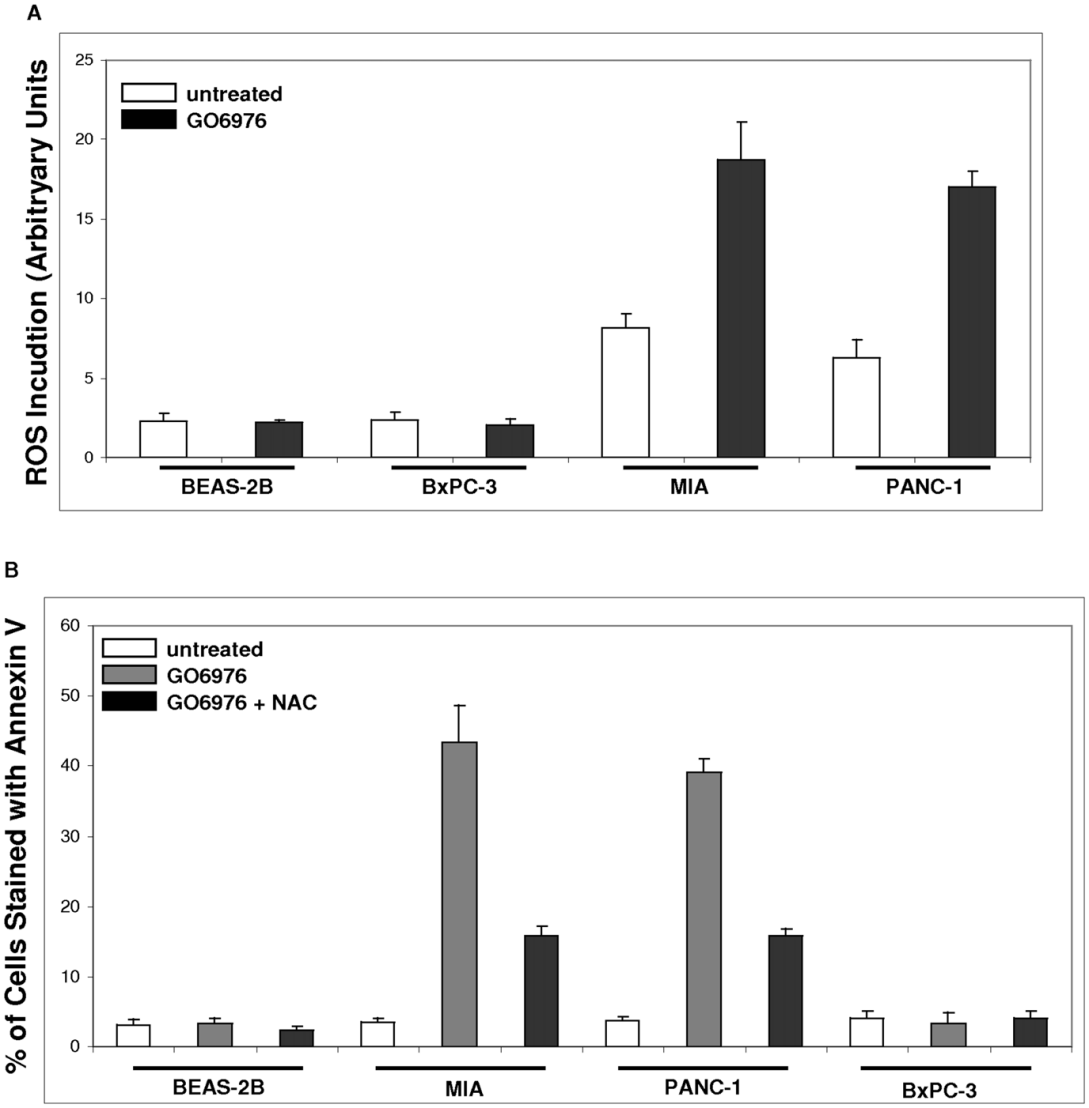
Upregulation of ROS and induction of apoptosis in pancreatic cancer cells. **A.** The cells were treated with GO6976 or co-treated with GO6976 plus NAC (2.5 mM). After being stained with DCF, the levels of ROS in the cells were analyzed by a flow cytometer. **B.** The cells were treated with GO6976 (1 µM) or GO6976 plus NAC. Subsequently, the samples were collected and subjected for annexin V assay. The error bars are SD from 5 independent experiments (n = 5, *ρ*<0.05).

ROS (reactive oxygen species) is known to be required for cell growth and on the contrary, a persistently increased in ROS was shown to be able to cause DNA damage or trigger apoptosis [36]. Studies revealed that ROS in cells expressing oncogenes (such as *ras*) were often augmented, which might be due to high metabolic needs of neoplastic growth [36]. ROS levels in pancreatic cancer and BEAS-2B cells were measured under normal growth conditions or after the treatment with GO6976 (**Fig. 4A**). A moderate amount of ROS was detected in MIA or PANC cells, which was dramatically elevated after the addition of GO6976. ROS was at baseline levels in untreated BxPC-3 or BEAS-2B cells and the treatment with the PKC inhibitor did not affect ROS production or accumulation.

To determine whether ROS played a role in the induction of apoptosis following PKC inhibition in pancreatic cancer cells expressing mutated *K-ras*, annexin V assay was conducted (**Fig. 4B**). Again, MIA and PANC-1 cells were sensitive to GO6976 treatment, which was partially, but significantly blocked by NAC (N-acetyl-L-cysteine, a ROS inhibitor). Since NAC did not completely suppressed apoptosis in these cells, it suggested the participation of other pathways in the execution of this cell death program. Consistently, pancreatic cancer cells without expressing mutated *K-ras* or human lung epithelial BEAS-2B cells were non-responsive to GO6976 as well as to the combination treatment of GO6976 plus NAC.

### Activation of Caspase 3 in this Lethal Reaction Occurred in MIA or PANC-1 Cells

**Figure 5:**
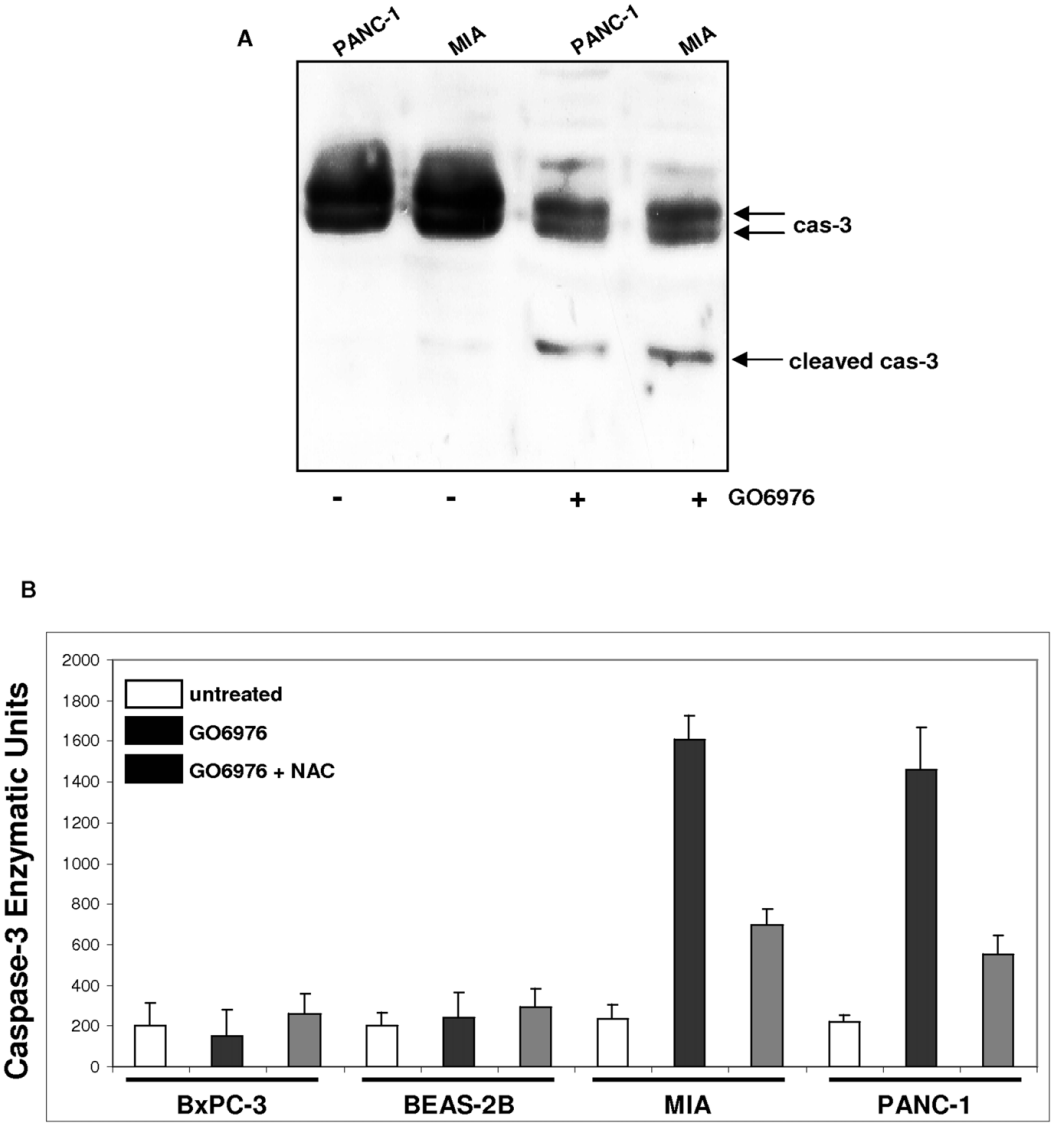
Caspase 3 activation in response to the suppression of PKC. **A.** With or without GO6976 treatment, cell lysates were prepared and immunoblotted with anti-caspase 3 antibody. **B.** The cells were subjected to the treatment with GO6976 or GO6976 plus NAC. Subsequently, annexin V assay was performed. The error bars represent SD from 3 independent experiments (n = 3, *ρ*<0.05).

Caspase family consists of more than 10 members [37]. In response to apoptotic stimuli, caspase-3 was shown to function as an executioner in caspase cascade for the completion of cell death program [37]. In this process, caspase-3 was required to be cleaved into a small, active fragment. To test the activation status of caspase 3, the presence of the cleaved caspase 3 in MIA or PANC-1 cells was analyzed by immunoblotting (**Fig. 5A**). Following the treatment with GO6976, a small, cleaved caspase-3 was indeed present in these two pancreatic cancer cells. The active form of this caspase was undetectable in GO6976-treated BxPC-3 or BEAS-2B cells (data not shown). To further examine the activation of caspase 3 under the same experimental setting, caspase 3 activity was analyzed (**Fig. 5B**). Following the treatment with the inhibitor, the activity of this protease was upregulated in MIA and PANC-1 cells, which was partially suppressed by adding NAC. Consistently, the activity of caspase 3 was absent in GO6976-treated BxPC-3 cells without expressing mutated *K-ras* or BEAS-2B cells. The results suggested that caspase 3 participated in the execution of the GO6976-induced apoptosis in pancreatic cancer cells harboring oncogenic *K-ras*.

### Upregulation of the Apoptotic Factor PUMA in a p73-dependent Fashion During GO6976-Induced Apoptosis

**Figure 6:**
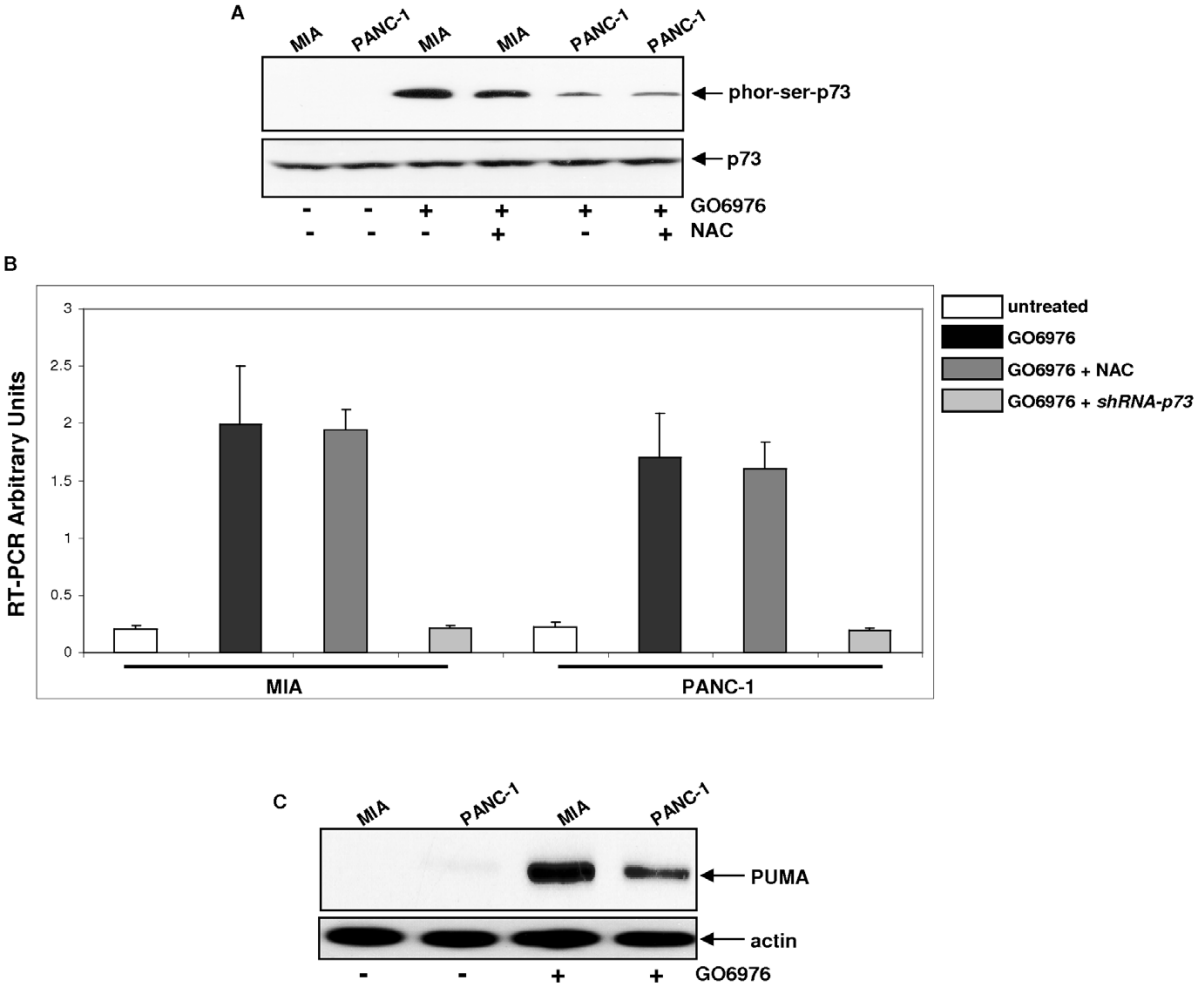
Phosphorylation of p73 and upregulation of PUMA in pancreatic cancer cells. **A.** With or without the treatment with GO6976 or GO6976 plus NAC, lysates were immunoprecipitated with anti-p73 antibody. The immunoprecipitates were then subjected to immunoblotting using the anti-phosphorylated serine antibody. The level of the immunoprecipitates was judged by re-probing the blot with anti-p73 antibody. **B.** Total RNAs from the cells with or without treated with GO6976, GO6976 plus NAC or GO6976 plus *shRNA-p73* infection were isolated. Equal amount of RNAs was reverse-transcribed, and the expression of *PUMA* was tested by RT-PCR. **C.** With or without GO6976 treatment, MIA or PNAC-1 cells were subjected to immunoblotting analysis for PUMA expression. Equal loading of total proteins were determined by rep-probing the blot with anti-β-actin antibody.

p73 belongs to p53 family and shares homology with p53 not only in their sequences, but also the transactivation, DNA-binding or oligomerization functions [38]. In the p73-mediated apoptosis, the apoptotic-related factor PUMA was often upregulated [38]. We previously reported that after PKC inhibition, p73 in murine fibroblasts ectopically expressing *Ha-ras* was activated and responsible for the initiation of apoptosis [28]. During this cell death process, p73 was phosphorylated at its serine residues [28]. To test whether p73 was phosphorylated in GO6976-treated MIA or PANC-1 cells, immunoblot analysis was conducted (**Fig. 6A**). The phosphorylation of p73 was occurred at its serine residues in the treated cells, but not in control cells. The GO6976-mediated p73 phosphorylation did not altered by the addition of NAC. Subsequently, the expression of p73-regulated *PUMA* gene in MIA or PANC-1 cells was examined by real-time PCR analysis (**Fig. 6B**). The level of the gene expression in the cells was significantly increased after the treatment with GO6976. The upregulation of the gene was again unchanged in the presence of NAC, but completely suppressed by the transient infection of *shRNA-p73*. Consistently, the protein expression was augmented after the treatment with the PKC inhibitor (**Fig. 6B**). The introduction of *shRNA-p73* into the cells also blocked the induction of PUMA protein, which was not affected by the addition of NAC (data not shown). It appeared that PUMA was involved in the axis of p73-mediated signaling in this apoptotic process.

### Cooperation of ROS and p73 for the Induction of GO6976-mediated Apoptosis

**Figure 7:**
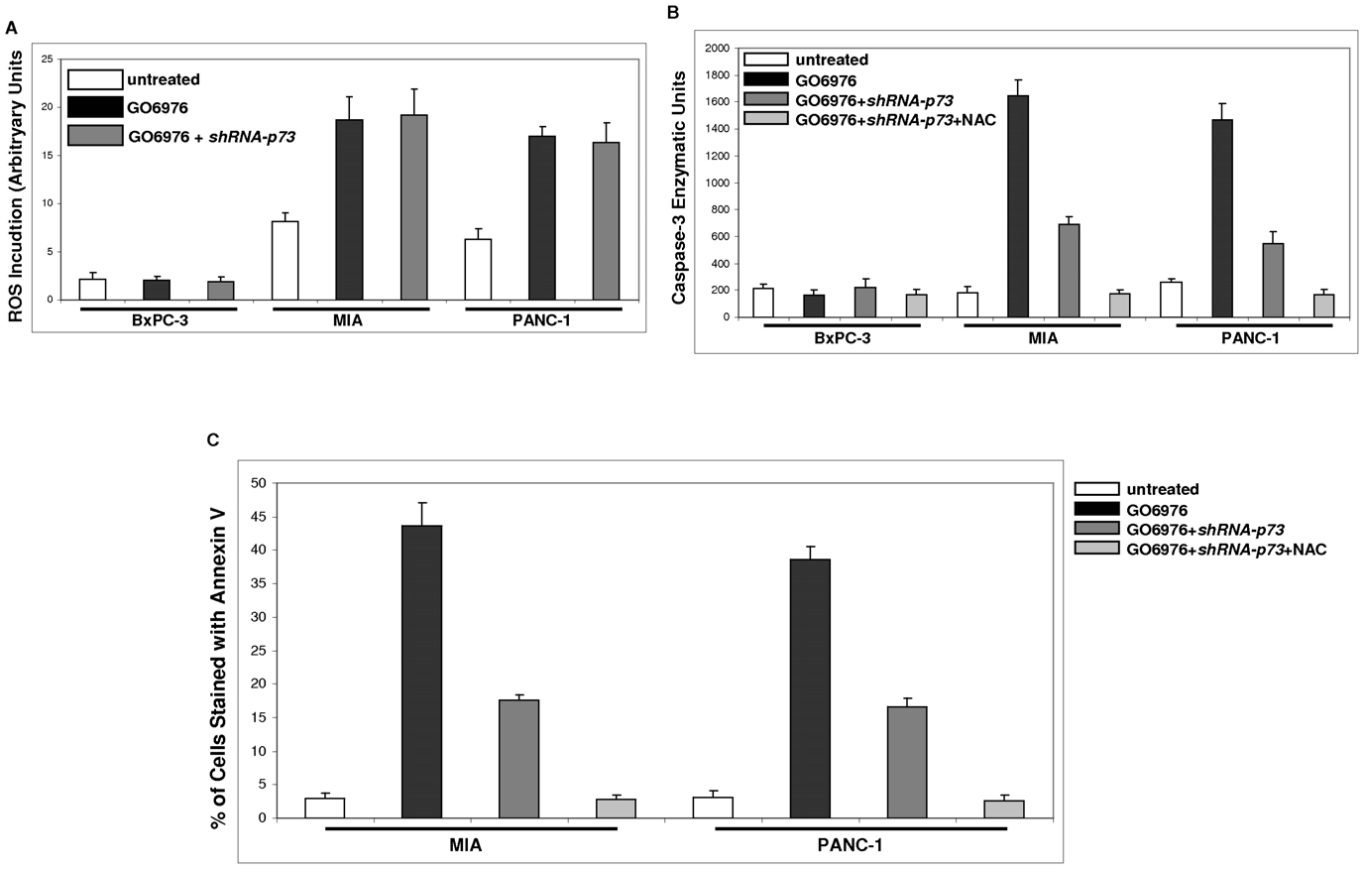
Induction of apoptosis in pancreatic cancer cells harboring mutated *K-ras*. A. The level of ROS in the cells treated with GO6976 or GO6976 plus *shRNA-p73* infection was analyzed. The error bars represent SD from 3 independent experiments (n = 3, *ρ*<0.05). **B.** The activity of caspase 3 in the cells treated with GO6976, GO6976 plus *shRNA-p73* infection or GO6976 plus NAC plus *shRNA-p73* infection was assayed. The error bars represent SD from 3 independent experiments (n = 3, *ρ*<0.05). **C.** Annexin V assay in MIA and PANC-1 cells treated with GO6976, GO6976 plus *shRNA-p73* infection or GO6976 plus NAC plus *shRNA-p73* infection was performed. The error bars represent SD from 3 independent experiments (n = 3, *ρ*<0.05).

It is well known that multiple, apoptotic signaling pathways are being integrated for a full execution of cell death program. Since ROS and p73 appeared taking part in the induction of apoptosis in GO6976-treated pancreatic cancer cells harboring mutated *K-ras*, we first tested if p73 played any roles in the upregulation of ROS in the pancreatic cancer cells (**Fig. 7A**). Again, a moderate among of ROS was detected in untreated MIA or PANC-1 cells, which was further increased after PKC inhibition. The transient infection of *shRNA-p73* had no influence on the induction of ROS, suggesting that p73 in our experimental setting was not involved in the ROS signaling. Subsequently, caspase-3 activity was analyzed after the knockdown of p73 or co-inhibition of p73 and ROS (**Fig. 7B**). The introduction of *shRNA-p73* partially blocked the activity of caspase-3 in GO-6976-treated MIA or PANC-1 and the activity of this protease was completely abolished after the co-suppression of p73 and ROS. The occurrence of apoptosis after the same treatments was then analyzed by annexin V assay (**Fig. 7C**). The knockdown of *p73* partially inhibited the GO6976-induced apoptotic process in MIA or PANC-1 cells. In the absence of both *p73* and ROS, GO6976 was unable to initiate apoptosis in these cells. The data suggested that at least two apoptotic pathways: p73 and ROS took part in this lethal reaction.

## Discussion

Pancreatic cancer is a devastating human malignancy and one of the leading causes of cancer death [1]. Prognosis of this disease is dismal, because of the lack of effective treatments. It is known that gain of functional mutations in *K-ras* occur at early stages in human pancreatic cancer patients or mouse models that were generated by knockout of tumor suppressor genes of *p16, Arf*, or *p53*, respectively or in combinations [12]–[14]. These findings indicate the importance of K-Ras in the genesis and development of pancreatic cancer. The goal of our study was to explore the new strategy targeting oncogenic Ras for pancreatic therapeutics. The present study demonstrated that aberrant, mutated *K-ras* was able to re-direct pancreatic cancer cells towards to apoptosis following the suppression of PKC. In this apoptotic process, multiple signaling pathways were involved. After the treatment with the PKC inhibitor, the level of ROS in the pancreatic cancer cells expressing mutated *K-ras* was increased, accompanied with the induction of apoptosis. However, the addition of NAC partially blocked the cell death process. p73 was phosphorylated and, PUMA gene and protein were upregulated. Furthermore, caspase 3 was cleaved for the execution of cell death program. The co-inhibition of ROS and p73 achieved an ultimate blockade effect on apoptosis in GO6976-treated pancreatic cancer cells harboring mutated *K-ras*. Thus, our study indicates that aberrant Ras, together with loss of PKC is synthetically lethal in the pancreatic cancer cells. The data also suggested a potential balance between Ras and PKC that determines the threshold of apoptosis in the cells.

Mutational activations of *ras* genes are one of the key events during the initiation and development of various types of human malignancies [15]. In the process of transformation, persistent increases in Ras activity switch on various downstream effector pathways, leading to phosphorylation chain reactions for the activation of pro-growth transcriptional factors [39]. Despite the essential involvement in cell growth and differentiation, hyperactive Ras, under certain circumstances, can be re-directed to apoptosis. Numerous studies have highlighted the roles of Ras in the regulation of apoptosis [20]. In particular, it has been observed that treatments with PKC inhibitors could induce apoptosis in murine fibroblasts or rat lung epithelial cells overexpressing oncogenic *ras* [22], [28]. Here, we demonstrate that PKC activity in the pancreatic cancer cells harboring mutated *K-ras* was slightly augmented, which might be necessary for coping with aberrantly high Ras activity for surviving. After PKC was suppressed, mutated K-Ras could not maintain high metabolic needs of the cancer cells and the lethal reaction was initiated. Since high percentages of human cancers harboring oncogenic *ras*, PKC appears an ideal intracellular target for the induction of apoptosis, with a low or none toxic effect on surrounding normal tissues or cells.

More than 11 serine/threonine protein kinases belong to PKC family, some of which are structurally distinct or functionally diverse. However, there is a functional redundancy among these PKC isozymes for restoring normal physiological status when one or more PKC isozymes are knocked out or nonfunctional. The roles of PKC isoforms in the regulation of cell growth or death are rather controversial, depending upon different types of cells or cellular contexts. For example, it has been suggested that PKC α, β, δ possessed duel roles in the regulation of cancer development or programmed cell death, indicating the complexity of these PKC isozymes [24]. Cross-talks among PKC isoforms and with other intracellular signal transducers were often present in a hierarchy order or different compartments to guide cells to achieve different outcomes. It was also shown that PKC and Ras signaling pathways were interconnected, especially in lymphocytes [24], [40]. Upon mitogenic stimulation, the SH2 binding sites of PKC were phosphorylated, which in turn recruits the Grb2/SOS complex and further activated Ras signaling in T lymphocytes [24]. PKC was reported to negatively regulated Ras pathway, through affecting its effector Rel [24]. In NIH3T3 cells ectopically expressing *v-Ha-ras*, p73 functioned downstream of PKCα and β and as a sensor to determine the threshold of apoptosis [22]. Using the PKC inhibitor GO6976 that inhibits the phorbol ester-dependent PKC isoforms, we in this study showed that the pancreatic cancer cells with hyperactive K-Ras could be efficiently sensitized to apoptosis. The investigation to identify key PKC isoform(s) in the regulation of this apoptotic process is under way.

ROS often acts as an intracellular signal transducer of growth factors [36]. Some mitogenic stimuli are able to augment the level of intracellular ROS, resulting in altering the structure of the cytoskeleton and further inducing transformation [36]. In PC12 cells, Ras was shown to upregulate ROS production upon EGF stimulation [41]. Studies also demonstrated that oncogenes (such as *myc* or *ras*), through persistently perturbing the state of intracellular redox, were able to cause chromosomal aberrations and subsequently disrupt genetic integrity to promote tumorigenesis [36]. Despite regulating cell proliferation and transformation, an increase in ROS was suggested to play an obligatory role in the induction of apoptosis [22]. In TNFα-induced programmed cell death, NF-κB and JNK cooperated to stimulate ROS production, leading to caspase cascade and mitochondrial depolarization [42]. Ectopic expression of *Ha-ras* in murine fibroblasts induced cell death through eliciting ER (endoplasmic reticulum) stress and activating UPR (unfolded protein response) [22]. The current study demonstrated that although ROS was moderately elevated in pancreatic cancer cells expressing mutated *K-ras* under normal growth conditions, inhibition of PKC severely disrupted the equilibrium of the redox state and induced a significant accumulation of ROS in the cells. Thus, PKC appears an important factor to maintain the homeostasis of pancreatic cancer cells harboring an aberrant *K-ras*.

p73 belongs to p53 family proteins and, shares the structural and functional homologue with p53 [38]. Studies showed that p73 played a significant role in DNA damage- or stress-induced apoptosis. Nuclear c-Abl was activated by genotoxic stress and further phosphorylated p73 for the induction of apoptosis [43]. In this cell death process, nuclear c-Abl interacted with p73 and further stimulated p73-regulated activities. It was also shown that in response to ionizing radiation or cisplatin treatment, p73 played a key role in the transmission of the apoptotic signaling [44]. In murine fibroblasts overexpressing *v-Ha-ras*, PKC δ interacted with and further activated p73 to trigger an apoptotic crisis [28]. In this study, we demonstrated that p73 was phosphorylated in pancreatic cancer cells harboring mutated *K-ras*, and subsequently upregulated the level of the expression of the apoptotic factor PUMA. The underlying mechanism by which PUMA participates in this apoptotic process remains to be further investigated.

In summary, genetic mutations in *K-ras* appear uniquely present in 90% of human pancreatic cancer lesions and this disease is one of the leading causes of cancer death. Thus, there is an urgent need for discoveries of effective treatments clinically. Our current study demonstrated that aberrant K-Ras could re-direct pancreatic cancer cells towards apoptosis, in which ROS and p73 appeared necessary for the initiation of this cell death program. Under normal growth conditions, gain or loss of function of either Ras or PKC alone is compatible with the viability of cells. However, when PKC activity or expression is suppressed, hyperactive K-Ras is unable to sustain the homeostasis, resulting in an apoptotic crisis. The data presented here provide a foundation for developing new therapeutic strategies that can kill pancreatic tumors harboring mutated *K-ras* at clinically achievable doses, with effective indices that are better than those of existing drugs.

## Materials and Methods

### Cells and Antibodies

Human pancreatic cancer BxPC-3, MIA, and PANC-1 cells were purchased from ATCC (American Type Culture Collection, Rockville, MD) and cultured in Dulbecco modified Eagle medium (DMEM) containing 10% newborn calf serum (Gibco, Gaithersburg, MD). Human lung epithelial cells were also purchased from ATCC and cultured in the medium according to the recommendation by the company. The *shRNA-p73* inserted in a lentiviral small-hairpin RNA expression vector was transiently infected into cells.

Anti-Ras, GST, PKC, caspase-3 and phor-p73 antibodies were purchased from Cell Signaling Technology.

### Measurement of Ras Activation

After the treatments, cells were lysed in a buffer containing 25 mM HEPES, pH 7.5, 150 mM NaCl, 0.25% sodium deoxycholate, 10% glycerol and 25 mM NaF. Proteins were normalized to 1 mg/ml, and precipitated by mixing with 20 µg of Raf-RBD overnight at 4°C. The complexes were washed and separated on a 10% SDS-PAGE gel and immunoblotted with an anti-pan-Ras antibody.

### Analysis of ROS

Treated or untreated cells were washed with ice-cold PBS and resuspended in 5 µg/ml of 2′, 7′-dichlorodihydrofluorescein diacetate (DCF) (Molecular Probes). The samples were incubated for 10 min at room temperature and analyzed immediately by a flow cytometer.

### Immunoblotting Analysis

After treatments, cells were lysed in the lysis buffer (50 mM Tris-HCl, pH 8.0, 150 mM NaCl, 1% Triton-X114, 0.5% sodium deoxycholate, 0.1% sodium dodecyl sulfate, containing 1 mM phenylmethylsulfonyl fluoride, 1 µg/ml aprotinin, 1 µg/ml leupeptin, 1 µg/ml pepstatin A) on ice for 30 min. The total protein concentrations in the cell lysates were normalized, separated on a 10% SDS-PAGE gel, and visualized by corresponding antibodies.

### PKC Enzymatic Assay

Following treatments, PKC activity in cell lysates was analyzed using PKC assay kit (Cell Signaling Technology) that contains a specific substrate peptide for PKC and an inhibitor mixture to block other serine/threonine kinases (such as the inhibitors of PKA and calmodulin-dependent kinases). Subsequently, ^32^P-incorporating substrates were separated from the residual [^32^P] ATP using p81 phosphocellulose papers and the radioactivity incorporated into the substrates was measured by a scintillation counter.

### Annexin V Assay

After treatments, cells (1 x 10^6^) were washed twice with cold PBS and stained with Annexin V-FITC using the Annexin kit (BD Biosciences) to detect apoptotic cells using a flow cytometer or counted 150 cells under a fluorescent microscope.

### Statistical Analysis

Three to five independent repeats were conducted in all experiments. Error bars represent these repeats. A Student’s T test was used and a *ρ* value of <0.05 was considered significant.
